# Educational and labor wastage of doctors in Mexico: towards the construction of a common methodology

**DOI:** 10.1186/1478-4491-3-3

**Published:** 2005-04-15

**Authors:** Gustavo Nigenda, José Arturo Ruiz, Rosa Bejarano

**Affiliations:** 1 Centre for Social and Economic Analysis in Health, Mexican Health Foundation, Mexico, Mexico

## Abstract

**Background:**

This paper addresses the problem of wastage of the qualified labor force, which takes place both during the education process and when trained personnel try to find jobs in the local market.

**Methods:**

Secondary sources were used, mainly the *Statistical yearbooks *of the National Association of Universities and Higher Education Institutions (ANUIES in Spanish). Also, the 2000 Population Census was used to estimate the different sources of labor market wastage. The formulas were modified to estimate educational and labor wastage rates.

**Results:**

Out of every 1000 students who started a medical training in 1996, over 20% were not able to finish the training by 2000. Furthermore, out of every 1000 graduates, 31% were not able to find a remunerated position in the labor market that would enable them to put into practice the abilities and capacities obtained at school. Important differences can be observed between generalists and specialists, as well as between men and women. In the case of specialists and men, lower wastage rates can be observed as compared to the wastage rates of generalists and women. A large percentage of women dedicate themselves exclusively to household duties, which in labor terms represents a wastage of their capacity to participate in the production of formal health services.

**Conclusion:**

Women are becoming a majority in most medical schools, yet their participation in the labor market does not reflect the same trend. Among men, policies should be formulated to incorporate doctors in the specific health field for which they were trained. Regarding women, specific policies should target those who are dedicated full-time to household activities in order to create the possibility of having them occupy a remunerated job if they are willing to do so. Reducing wastage at both the educational and labor levels should improve the capacity of social investment, thereby increasing the capacity of the health system as a whole to provide services, particularly to those populations who are most in need.

## Introduction

The medical workforce has been studied from diverse points of view by means of a wide variety of methodologies and at varying levels of depth. Some studies approach the subject from the formal education standpoint, examining the problems encountered in the updating of knowledge, the number of human resources available and the distribution and proportion of resources according to the population. Other studies look into working conditions, prevailing ethical codes and productivity, among other topics. In a first review of the available specialized literature, very few studies were found to deal systematically with the difficulty for trained individuals in the health field to put into practice their acquired knowledge to deliver services [1].

In many countries the supply of doctors is being generated without planning and, very frequently, with no regulation at all. Doctors are being educated in schools that provide them with a professional degree; in spite of this, they will eventually face difficulties in finding a job. This problem is related to the qualified labor force wastage, as well as to the specific market configuration. On the other hand, there is another source of wastage that takes place during the education process and is reflected in the demand for medical education, the number of dropouts, the final efficiency rates at a national level and the organization and location of medical schools [2].

Besides these problems, the wastage of scholars has various facets that are not easy to identify, study or solve. One of these, perhaps the most important, is the enormous economic burden for a society represented by an inconclusive education process in which many students drop out before finishing their studies, making it impossible for them to put into practice the knowledge they acquire. This represents a fruitless investment and a wastage of economic resources that no nation can afford, particularly in the developing world.

Social investment in medical training should produce a benefit for societies, their institutions and citizens. The role of medical practice is highly valued in most societies, but it is up to governments to establish regulations that enable education to produce well-trained doctors practising under high ethical and professional standards. Government intervention is crucial to guaranteeing that this social investment provides the highest possible outcome. Health care reforms are taking place in many countries and are creating new conditions for medical practice, but not many of them regard HRH as a strategic planning issue [3].

It is also necessary to consider that wastage and geographical distribution are very much related. Indeed, doctors in Mexico have been concentrated for many years in urban areas, yet these areas also show high underemployment and unemployment rates. On the other hand, the lack of interest of doctors to practice in remote regions paradoxically results in a very reduced wastage in suburban and rural areas [4].

Therefore, the dynamics between human resources supply and the actual requirements of institutions lead to breakdowns that are expressed as labor wastage – that is to say, time and capabilities that graduate doctors do not put into practice either directly or indirectly for the production of health services.

The paper is not limited to the estimation of wastage rates but, based on the information obtained, widens its scope to discuss the implications for HRH – among them, the need to solve wastage in urban areas while encouraging the flow of doctors to remote areas. It also suggests the need to protect social investment in medical education. A further issue emphasized is the participation of women, as it has been made evident that their enrolment to medical schools has grown significantly in the last 15 years, yet their engagement in the labor market still occurs under different conditions as compared to those of men [5].

## Objectives

The paper pursues the following objectives:

• to characterize the problem of wastage of doctors during their education process and in the labor market;

• to begin to construct a methodology that allows for the study of the problem in Mexico and, at the same time, could be replicated in other countries;

• to contribute to the discussion of the wastage of human resources in the health sector at the national and international levels;

• following the initial diagnosis about the wastage found in medical personnel, to use the sex variable to explore a relevant dimension of the problem.

## Conceptual framework

The purpose of this section is to establish definitions that will set out the foundations for the presentation of results based on the information gathered. Due to the particularities of each field and for the sake of the exposition, the educational and labor market subjects will be addressed separately.

### Wastage during education

Many studies in Mexico have dealt with the problems of school dropouts, final efficiency, repetition, exclusion and effectiveness and efficiency of the training institutions, among others. However, both at national and international levels little has been done to study the problem of specific professions (such as medicine) comprehensively so as to fully understand the causes and effects of the wastage of human and economic resources during the educational process [6].

The data presented in the following sections were obtained mainly from the National Association of Universities and Higher Education Institutions (ANUIES in Spanish), a nongovernmental agency that for more than 35 years has been responsible for compiling and systematizing information provided by 138 public and private institutions [7]. The stability and duration of this process of data collection renders the information obtained from this source highly reliable.

The following concepts have been taken from the existing studies:

### Global attrition

This is a condition experienced by someone who does not comply with the timelines and does not complete the corresponding stages of the study plan of an institution in a specific year [8]. The student voluntarily or involuntarily interrupts his/her studies without having completed the studies required for his or her career. Such an interruption is not a spontaneous act, as there are family, social and institutional factors behind it [9]. Among the main causes of an interruption of studies are family attitude, economic conditions, study habits, inadequate selection of career, motivation, age, civil status and employment [10].

### Graduates

Students who have completed the total number of credits required and/or have fully complied with the established procedures (such as writing a thesis) included in the plan of studies, receive a degree from a university or other institution of higher education.

### Wastage in the labor market

There are factors affecting the labor market that are difficult to identify and much more difficult to quantify. The issue of unemployment and its varied manifestations in shape and time make it a somewhat polemical topic when dealing with secular variations. Although parameters to measure unemployment have been established at the international level, this has not allowed to count on timely information that might be comparable among countries and even among regions and states within a country [11]. In the case of professional groups, quantifying unemployment is not enough to understand labor market unbalances.

In the case of Mexico, the National Institute of Statistics, Geography and Informatics – INEGI – carries out two important data collections reporting results on various aspects related to employment at the national level: the General Census on Population and Housing is carried out every ten years, and a National Survey of Urban Employment, every three months.

Periodically INEGI reports a series of statistics about the employment situation across the country; one of these is the rate of open unemployment at the national level. However, this rate alone is not the most adequate indicator to establish the dimension of labor wastage, among other reasons because it is a macro indicator that shadows other levels of participation in the labor market that could be considered inadequate for an individual with professional training.

As part of the conceptual and methodological definitions of the present work we propose to estimate the rate of wastage among doctors, which would incorporate all those conditions in which a graduate from a medical school does not put into practice the knowledge gained from the school for the production of health services. Some situations, such as unemployment, are easy to identify as part of this wastage, but others (e.g., that of individuals who work less than 20 hours a week) are more difficult. We quantify wastage through the number of individuals who fall into categories in which their training does not match their labor activity. (Table 1)

**Table 1:** Sources of training and labor wastage. Categories used to estimate wastage in training are: attrition and non-graduated. Categories used to estimate labor wastage are: unemployment, household activities, and other jobs (working in activities not related to the field of training).

Training wastage*		
Attrition	Non-graduate	
**Labor wastage**		
Unemployment	Household	Other jobs (working in activities foreign to the field of training)

* Those who temporarily withdraw from studying and those who return to study are not included.

For the purpose of this study, the following concepts would need to be taken into account [12].

### Employment

This is the situation in which graduates work as general practitioners or, as students for a medical specialization degree, in a full-time clinical practice at a hospital. It also includes specialist doctors with a labor position in health institutions according to the degree obtained. The category also comprises those doctors who are dedicated to research and/or teaching activities and those in managerial positions in health institutions.

### Unemployment

This refers to individuals without employment, including those awaiting a reply about a job application (and who are not looking for any other job), those who are too discouraged to continue looking for a job and those who are actively seeking one.

### Underemployment

This refers to individuals who have finished their studies and carry out activities different from their training; such activities may take place outside the health sector and/or in areas not directly related to the delivery of health services. To estimate underemployment in the general population, labor specialists normally consider working time and income criteria. In this case, since we are dealing with a professional group, a dimension that has proved to be appropriate for this measurement is the match between training and labor activities. This type of underemployment is known as qualitative underemployment and it is useful to understand the participation of highly trained groups of the population.

### Household activities

These refer to individuals who do not have remunerated work because they are dedicated to household activities on a full-time basis.

### Inactive, not available

This refers to groups of individuals who are retired, pensioned or suffer a permanent disability.

### Labor wastage

This refers to qualified human resources who do not practice activities related to their formal education because they are not employed (including those dedicated to household activities) or because they carry out activities that do not correspond to their training.

## Methods

With respect to wastage that takes place during the years of study, the *Anuario Estadístico *(Annual Statistical Book) published by ANUIES between 1976 and 2001 was used as the main source of information. It was necessary to carry out our own calculations to estimate enrolment, incoming students, graduates and abandonment per group pertaining to a common period of study, with a cohort of five years each.

Since there is no information on the incoming students disaggregated by sex, drop-outs and graduates for the years before 1996, it was possible to calculate rates of abandonment and final efficiency for only two graduating classes.

To calculate the wastage in the education of the medical students, the following formulas were established:



As to the wastage in the labor market, the database of the *XII Censo General de Población y Vivienda, 2000 *[13] (XII General Census on Population and Housing, 2000) was examined for information about the following variables: sex, age, education and individuals who had studied for the career of medicine. The information about specialists available in the Census database was very limited. Thus, we decided to focus our exercise on generalists (in Mexico, generalists are those graduated from medical schools who have not obtained a specialist degree).

As to the latter, census codes were compared to find out their activity status (their own or activities not related to their education), occupation (making it explicit if they were dedicated to household activities), and whether they were unemployed, retired, pensioned or permanently disabled.

Codes used to classify the activity area were taken from the North American Industry Classification System (NAICS) used by the 2000 National Census of Population and Housing. Category 6 corresponds to health services. Codes used to classify the educational level correspond to a classification developed by the National Institute of Statistics, Geography and Informatics.

Once this information was processed, the following formulas were built. To calculate the rate of employment among individuals who studied medicine:



For the rate of unemployment, the formula used was:



And for the rate of wastage it was established that:



## Results and discussion

Based on the definitions set forth in the framework provided for this work and the formulas set out in the methodology section, the following results for the total medical personnel were obtained.

### Wastage during education

In general, enrolment in the career of medicine in Mexico has shown non-linear behavior during the last 25 years. At the beginning of the 1990s it showed a tendency to drop, mainly because of the official policy implemented in the mid-1980s to try to halt the high demand then evident. Despite this, on average enrolment grew by 22.8% during the period 1990–2001 (Table 2).

**Table 2:** Total enrolment, medical schools in Mexico, 1990–2001. In general, enrolment in the career of medicine in Mexico has shown non-linear behavior for the past 25 years. It was at the beginning of the 1990s when it showed a downward trend, mainly because of the official policy implemented in the mid-1980s to halt the high demand to enrol that was evident at that time. Yet on average, enrolment increased by 22.8% during the 1990–2001 period.

**Year**	1977	1980	1983	1986	1990	1993	1996	1999	2001
**Total**	85 822	77 474	76 424	64 853	57 667	55 591	59 645	64 594	70 830

Source: ANUIES, Anuarios estadísticos, 1990–2001

**Figure 1:**
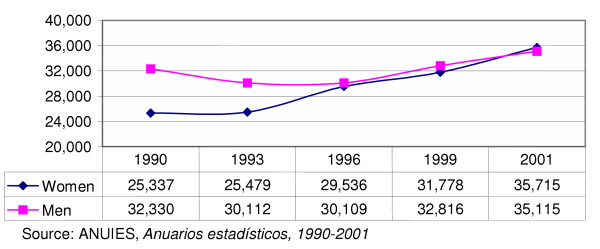
**Total enrolment in the career of medicine by sex, 1990–2001**. Throughout the period, the proportional participation of women maintained a constant growth. According to the annual statistical book from ANUIES, the percentage of women enrolled in medicine jumped from 43.9% in 1990 to 50.4% in 2001. It 1999 the number of women enrolled outnumbered men for the first time, by 1038.

Throughout this period, the proportional participation of women maintained constant growth. According to the annual statistical book from ANUIES, the percentage of women enrolled in medicine jumped from 43.9% in 1990 to 50.4% in the year 2001. It was in the year 1999 when the women enrolled outnumbered men for the first time, by 1038 students (Figure 1).

**Figure 2:**
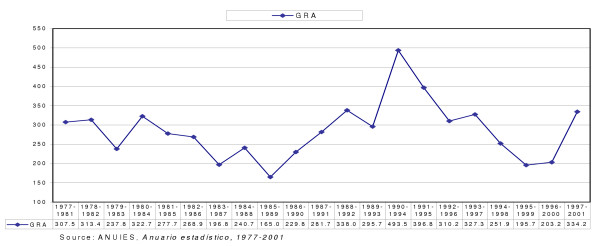
**Global rate of attrition (GRA) in medicine by group pertaining to the same period of study, 1977–2001**. To calculate the attrition in the medical profession, a series was constructed for each graduating group, from the first admission in 1977 up to the 1997–2001 graduating class. Once the series was completed, it could be established that the lowest level of drop-outs took place during the period 1985–1989, with a rate of 165.0 per thousand students, while the highest level was registered in the 1990–1994 class, with a rate of 493.5.

**Figure 3:**
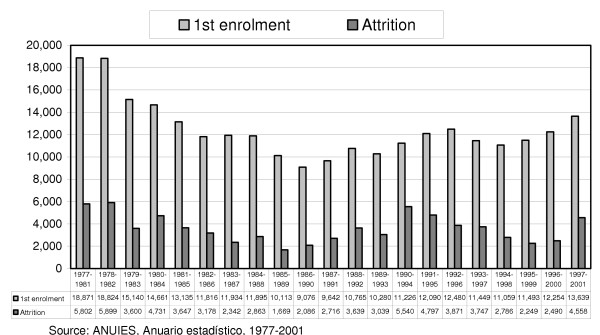
**Incoming students and attrition by cohort, 1977–2001**. An indicator that allows us to determine the wastage is the rate of final efficiency in the career of medicine at the national level. To this purpose, a series of graduating classes with the same cohort was constructed; this led to the identification of the highest final efficiency, which was achieved in the 1985 and the 1995 graduating classes, with a rate of 834.9 and 804.3, respectively. The second-highest rate was reached during the period 1995–1999.

An indicator that clearly illustrates wastage during education is the rate of final efficiency in the career of medicine at a national level. In the same manner, the series of graduating classes with the same cohort was constructed, enabling us to observe that the highest final efficiency was achieved in the graduating class of 1985 and that of 1995, with a rate of 834.9 and 804.3, respectively. After ten years the second-highest rate was reached, during the period 1995–1999 (Figures 2 and 3).

Although it is true that the final efficiency varies from one medical school to another (which also would be important to further investigate), there is no doubt that at the national level the registered rates are worrisome, given the number of medical students who do not complete their studies.

On the other hand, the proportion of incoming students in relation to the total enrolment for medical training in Mexico during the period 1977–2001 was calculated. The result for the first year (1977) was 21.9, and for the second (2001), 21.8. The similarity between these two years is peculiar, since nothing similar is observed for the remaining years. In fact, this proportion decreases at times (13.9 and 14.0 in 1986 and 1982), while at some others it increases (as in the years of 1997 and 1998, with 23.6 and 23.3, respectively).

The notoriously high rate of attrition in 1990–1994 may well be related to the economic crisis Mexico was facing by the end of the period, which made it very difficult for students and their families to afford medical education. The rate of efficiency shown in Figure 2 mirrors the capacity of schools, students and families to reduce the volume of drop-outs [14].

Figure 3 presents another way to express wastage in medical education. The proportion of drop-outs in the period 1990–1994 is the highest of all periods (50%). The volume of drop-outs in that period was similar to those of 1977–1981 and 1978–1982, but the volume of new enrolments in the latter two periods was 40% higher.

As can been seen in Table 3, the information about drop-outs, graduates and final efficiency does not show any significant difference whenever the sex variable is included. However, given that it was possible to obtain information by sex for only two classes pertaining to the same period of study, it would be difficult to reach any solid conclusion in this respect.

**Table 3:** Global rates of attrition and final efficiency of the medical graduates per thousand students by sex and groups pertaining to the 1996–2000 and 1997–2001 periods. The information about drop-outs, graduates and final efficiency does not show any significant difference when the sex variable is included. However, given that it was possible to obtain information by sex for only two classes for the same period of study, it would be difficult to draw any final conclusions regarding this issue.

**Cohort**	**Incoming students**		**Attrition**		**Graduate students**		**Global rate of attrition × thousand students**		**Rate of final efficiency × thousand students**	
	
	M	W	M	W	M	W	M	W	M	W
1996–2000	6 200	6 054	1 390	1 100	4 810	4 954	224.2	181.7	775.8	818.3
1997–2001	6 819	6 820	2 215	2 343	4 604	4 477	324.8	343.5	675.2	656.5

Source: ANUIES, Anuario estadístico, 1996–2001

* No data are available for 2002.

Upon constructing series of data by year, it was found that women have moved from representing 19% of the graduates in 1970 to almost half the graduates in the year 2001 (49.3%). Since 1996, the number of female medical graduates has been very similar to that of men. Something similar occurs when comparing the information about incoming students and dropouts (Table 4). The number of graduates who received their degree has not shown significant changes in the recent years: in 1996, 45.9% were women, a figure that went up to 49.3% in the year 2001.

**Table 4:** Incoming students, drop-outs and graduate students in medicine by year and sex, 1996–2002. Since 1996, the number of women medical graduates has been very similar to that of men. Something similar occurs when comparing the information about incoming students and drop-outs. The number of graduate students receiving their degree has not shown significant changes in recent years: in 1996, 45.9% were women, while in 2001 this proportion was 49.3%.

**Year**	**Incoming students**		**Percentage of total attrition**		**Percentage of total graduate students**	
	
	**Men**	**Women**	**Men**	**Women**	**Men**	**Women**
1996	6 200	6 054	47	53	51	49
1997	6 819	6 820	49	51	51	49
1998	7 456	7 064	53	47	50	50
1999	7 331	7248	51	49	50	50
2000	7 655	7 858	51	49	49	51
2001	7 501	7 962	45	55	51	49
2002	7 746	8 631	n/a	n/a	n/a	n/a

Source: ANUIES, Anuario estadístico, 1996–2002

* No data are available for 2002.

### Wastage in the labor market

Out of the total number of general physicians and specialists in 2000, 70% were working in medical care and 5% were studying. From a sex perspective, 13% of the men were not working and 2% of the women were inactive, while the percentage of men and women working in medicine was 75% and 59%, respectively; that is to say, there was a difference of 16 points favoring the number of men employed.

Out of the population with education in general medicine that was dedicated to household activities, only 0.5% were men while 11% were women. Another item of information that stands out regards inactive and unavailable personnel: in fact, there are more men than women as such, which may indicate that there are fewer women retired and pensioned [15] (Table 5).

**Table 5:** Occupational status of physicians by sex, 2000. Out of the population with education in general medicine who were dedicated to household activities, only 1% were men, while 12% were women. It should also be noted that more men than women are inactive and not available; this may indicate that there are fewer women retired and pensioned.

	**Total**	**%**	**Men**	**%**	**Women**	**%**
National total	204 778	100	133 673	65	71 105	35
Employed	142 923	70	100 818	75	42 105	59
Studying	10 122	5	4 596	3	5 526	8
Unemployed	10 892	5	5 385	5.5	5 507	8
Dedicated to household activities	7 895	4	8	0.5	7 887	11
Working in activities not related to the field of training	26 733	13	18 289	13	8 444	12
Inactive, unavailable	6 213	3	4 577	3	1 636	2

Source: Data generated by FUNSALUD from the XII General Census on Population and Housing, 2000.

Results after repeating the same operations, but taking into account the variable sex, are presented in Tables 6, 7 and 8.

**Table 6:** Rate of employment, unemployment and wastage, 2000. To estimate the rate of employment, the following categories were used: total of employed divided by the total number of doctors minus students and inactive. In the case of unemployment, the following formula was used: unemployment divided by employed plus other jobs plus underemployed. In the case of wastage, the formula used was: unemployed plus household plus other jobs divided by the total number of doctors minus students and inactive.

Rate of employment = A/ (T - B - F)	
	

Source: Data generated by FUNSALUD from the *XII *General Census on Population and Housing, 2000.

Where: A = Employment, B = Studying, C = Unemployed, D = Household, E = Others jobs, F = Inactive, T = Total

**Table 7:** Rates of employment in the health sector, employed in non-health activities and studying, 2000. Data used to estimate the rate of employment within the health sector were the following: the total number of employed divided by the total number of doctors minus the inactive. The formula used to estimate the students was: total number of students divided by the total number of doctors minus the inactive. To estimate the number of doctors working outside the health sector the following formula was used: other jobs outside the health sector divided by the total number of doctors minus the inactive.

Rate of employment in the health sector = A/T-F	
	

Source: Data generated by FUNSALUD from the *XII *I General Census on Population and Housing, 2000.

Where: A = Employment, B = Studying, C = Unemployed, D = Household, E = Others jobs, F = Inactive, T = Total

**Table 8:** Rates of labor participation (× thousand doctors) by sex, 2000. To estimate the rates of labor participation by sex, men and women constituted separate groups. Formulas were estimated in the same way as for the total population.

**Rates**	**Women**	**Men**
Rate of employment		
Rate of unemployment		
Rate of wastage		

Source: Data generated by FUNSALUD from the XII General Census on Population and Housing, 2000.

## Discussion

The methodology proposed in the present document can be reproduced in other countries if a population census database is available. WHO should encourage countries to estimate medical and other occupational groups' wastage rates in order to find out ways to reduce this phenomenon and to make the most of social and private investments [16,17].

In Mexico, an Inter-institutional Commission for the Training of Human Resources for Health, an entity created by high educational and health authorities to plan the supply, demand and distribution of human resources for health in the country, has been working for almost 30 years. It is suggested that the Mexican case be revised, since this might contribute to encouraging linkage and interaction between training and health services institutions with the aim of achieving a better planning process according to country-specific characteristics.

**Figure 4:**
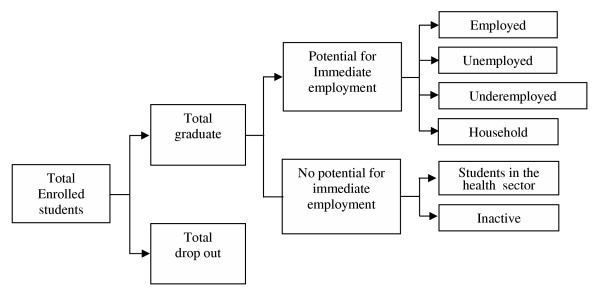
**Possible outcomes for individuals, from medical schools to the labor market**. The diagram shows that doctors enrolled at medical schools can follow one of two patterns: the first refers to students obtaining their diploma, and the second, to students dropping out or not fulfilling the established requirements to graduate. Once in the labor market, graduates can be divided into two subgroups: those who are ready to be immediately employed and those who are not. In turn, those who are ready can be employed, unemployed, underemployed or fully dedicated to household activities. Those who are not ready to be immediately employed are divided into students that go on for a specialization degree and those who are inactive. A proportion of those initially enrolled will later be represented by the rate of attrition.

Figure 4 shows that doctors enrolled at medical schools can follow one of two patterns: the first refers to students obtaining their diploma, and the second, to students dropping out or not fulfilling the established requirements to graduate. Once in the labor market, graduates can be divided into two subgroups: those who are ready to be immediately employed and those who are not. In turn, those who are ready can be employed, unemployed, underemployed or fully dedicated to household activities. Those who are not ready to be immediately employed are divided into students that go on for a specialization degree and those who are inactive.

The wastage of resources during the education of medical students is significant, as was shown in the results. This problem should be studied in further detail to enable us to arrive at an estimation of the economic cost that this represents at the individual, family and social levels. This economic cost cannot be estimated from the available information regarding wastage in medical training and in the labor market. Before moving ahead to the next step, it would be important to conceptualize the factors that intervene in the wastage process in order to attain an objective assessment of it. Questions such as how to determine and estimate indirect costs and how to assign a money value to the time lost when people abandon the market temporarily seem to be of utmost importance.

The inequalities between men and women in the labor market are reflected not only as higher wastage rates, as has been shown in this document. In this respect, there is an increasing trend toward a decline in working conditions imposed in many developing countries by employers, both private and public. This process of decline includes the stagnation of salaries, the lack of guarantee of labor rights and benefits, and the use of temporary contracts instead of permanent positions. In Latin America it is possible to identify countries where declining working conditions have spread, such as Argentina, Brazil, Ecuador, Mexico and Peru. Contracts reflecting these conditions are offered mostly to women [18].

In Mexico previous studies have shown a relation between geographical distribution and wastage. The highest levels of medical unemployment and labor wastage in Mexico are found in urban areas [19]. This fact can be appreciated when comparing states with large rural populations to states with more urbanized populations [20]. Within the first group the estimated availability of doctors per population tends to be lower than in the second group. For example, in the southern state of Chiapas, with 54% of its population being rural, the number of available doctors per 1000 inhabitants is 0.79, while in the Federal District (Mexico City), with 99.8% of its population being urban, the number of doctors per 1000 inhabitants is 3.03, 3.8 times higher than in Chiapas.

It would be a sound idea to sensitize medical students about the problem and the need to move on to unsaturated areas. On the other hand, health policies should enhance incentives for doctors to move to underserved areas, including higher salaries and the possibility of further training for those doctors who show willingness to take that option.

The top decision-making levels of the health system should be supporting the design and development of studies aiming at understanding in detail the issues concerning labor wastage; this would contribute to producing policy recommendations that stress the need for a comprehensive and coordinated institutional participation [21].

The methodology followed to calculate the wastage during the educational process as well as in the labor market was demonstrated to be adequate to support these kinds of studies. Hence, based on the information derived from a population census and on the management of similar variables, it would be possible to replicate this method in other countries. Additionally, this would make it possible to carry out comparative and complementary studies that would set forth the problem in more detail and would assist in the formulation of alternative policies within the health sector.

Such a methodology can be applied without further implications for the exploration of the development and labor conditions that prevail in other occupational categories, such as nursing and dentistry.

## Conclusion

Although assessment of the wastage phenomenon could be more accurate if a wider set of data were available, it can in any case be said that in Mexico the wastage of human resources in the health sector is a major problem. In the year 2000, 310 of every 1000 enrolled students did not finish their training. This represents an important source of wastage of human resources.

There is no doubt that the wastage of a highly qualified workforce has a negative impact on the economy of any country. Governments and families invest huge amounts of material and financial resources to train professionals; ultimately these professionals may not find a position within the labor market in which the functions they perform accord with the long training they have received [22]. By the end of the 1990s, 720 of every 1000 enrolled medical students finished their training, and of 1000 doctors available in the labor market, only 58% found paid employment that enabled them to practise the skills obtained during their training.

As part of the problem, the experience individuals have accumulated to fully integrate into the labor market as well as the barriers and opportunities they face to get a job must be taken into account. Finally, health systems, including their educational components, must look for ways to reduce the wastage of human resources in order to increase the efficiency of the system as a whole; this should be considered a social imperative.

Unemployment and the rate of wastage among women are much higher than for men [23]. This reflects an inequitable labor situation that adds up to a series of disadvantages related to the male-centered social structure prevalent in Mexico. For example, men generally receive a higher income for carrying out the same tasks as women; they also are usually accorded administrative positions [24].

It is clear that the issue explored in the present article represents one of the faces of a long-standing paradox. The number of doctors in urban areas has surpassed the demand from the population to the point of producing unemployment, underemployment and, ultimately, wastage. In rural areas, in contrast, doctors are still absent in many communities, so people become ill and die of communicable and preventable diseases for want of competent medical care [25]. Understanding this phenomenon is the first step towards solving the problem. But to proceed with the second step, it will be necessary to develop new policies, based on sound information, aiming at attaining a more equitable and efficient distribution of resources, including health personnel.

## Competing interests

The author(s) declare that they have no competing interests.

## Authors' contributions

GN conceived the original idea of the paper; wrote some its sections, generated the conclusions and recommendations and corrected the whole of its contents. JAR wrote some sections of the paper and generated the conclusions and recommendations. RB wrote some sections of the paper, compiled the information and generated tables and graphs.

## References

[B1] Gupta N, Zurn P, Diallo K, Dal Poz MR (2003). Uses of population census data for monitoring geographical imbalance in the health workforce: snapshots from three developing countries. International Journal for Equity in Health.

[B2] Kolehmainen-Aitken R-L (2003). Decentralization's impact on the health workforce: Perspectives or managers, workers and national leaders. Boston: Joint Learning Initiative Working Group on Demand.

[B3] Rigoli F, Dussault G (2003). The interface between health sector reform and human resources in health. Human Resources for Health.

[B4] Wibulpolprasert S (1999). Inequitable distribution of doctors. Can it be solved?. Human Resources for Health Development Journal.

[B5] Knaul F, Frenk J, Aguilar AM (2000). The gender composition of the medical profession in México: implications for employment patterns and physician labor supply. Journal of American Medical Women's Association.

[B6] Arroyo Laguna J, Cuevas Álvarez L, Brito P (2002). Situación y desafíos de los recursos humanos en salud en el área andina. Presente y futuro en la formación, práctica y regulación profesional en ciencias de la salud.

[B7] Asociación Nacional de Universidades e Instituciones de Educación Superior Anuario Estadístico. México.

[B8] Martínez Rizo F (2001). Estudio de la eficiencia en cohortes aparentes. Deserción, rezago y eficiencia terminal en las instituciones de educación superior Propuesta metodológica para su estudio.

[B9] Durán J, Díaz G (1990). Análisis de la deserción estudiantil en la UAM. Mimeo.

[B10] Casares Ortiz R (1994). Exploración preliminar de la causalidad de la deserción de la Facultad de Medicina de la Universidad Autónoma de Yucatán. Educación y Ciencia.

[B11] Dovlo D (2003). Assessing HRH wastage and improving staff retention: an African perspective. Boston: Joint Learning Initiative Working Group onDemand.

[B12] Frenk J, Alagon J, Nigenda G, Muñoz G, Robledo C, Vazquez LA, Ramirez C (1991). Patterns of medical employment: A survey of imbalances in urban Mexico. American Journal Public Health.

[B13] Instituto Nacional de Estadística, Geografía e Informática (2001). XII Censo General de Población y Vivienda 2000. México.

[B14] Ruiz JA, Molina J, Nigenda G (2003). La formación de médicos y mercado de trabajo en México. Caleidoscopio de la Salud.

[B15] Michel JB (1984). Why do women physicians work fewer hours than men physicians?. Inquiry.

[B16] De Oliveira O, García B (1994). Trabajo femenino y vida familiar en México.

[B17] Phillip RK, Marder WD, Silberger AB (1990). The growing proportion of female physicians implications for US physicians supply. American Journal Public Health.

[B18] Langer A, Nigenda G (2004). Sexual and reproductive health and health sector reform in Latin America and the Caribbean.

[B19] Frenk J, Knaul FM, Vázquez-Segovia LA, Nigenda G (1999). Trends in medical employment: persistent imbalances in urban Mexico. American Journal Public Health.

[B20] Wibulpolprasert S, Pengpaiboon P (2003). Integrated strategies to tackle the inequitable distribution of doctors in Thailand: four decades of experience. Human Resources for Health.

[B21] Harrison ME (1998). Female physicians in Mexico: migration and mobility in the life course. Social Science and Medicine.

[B22] Weisman C, Teitelbaum M (1984). The work-family role system and physicians productivity. Journal Health Society Behavior.

[B23] Uhlenberg P, Cooney T (1990). Male and female physicians: family and career comparisons. Social Science and Medicine.

[B24] Phillip RK, Marder WD, Silberger AB (1990). The growing proportion of female physicians implications for US physicians supply. American Journal Public Health.

[B25] Nigenda G (1994). Los recursos humanos para la salud en busca del equilibrio.

